# Abrupt emergence of a large pockmark field in the German Bight, southeastern North Sea

**DOI:** 10.1038/s41598-017-05536-1

**Published:** 2017-07-11

**Authors:** Knut Krämer, Peter Holler, Gabriel Herbst, Alexander Bratek, Soeren Ahmerkamp, Andreas Neumann, Alexander Bartholomä, Justus E. E. van Beusekom, Moritz Holtappels, Christian Winter

**Affiliations:** 1 University of Bremen, MARUM – Center for Marine Environmental Sciences, Bremen, 28359 Germany; 2 Senckenberg am Meer, Marine Research Department, Wilhelmshaven, 26382 Germany; 3 Helmholtz-Zentrum Geesthacht, Institute for Coastal Research, Geesthacht, 21502 Germany; 4 University of Hamburg, Hamburg, 20146 Germany; 5 Max-Planck-Institute for Marine Microbiology, Bremen, 28359 Germany; 6 Alfred Wegener Institute, Helmholtz Center for Polar- and Marine Research, Bremerhaven, 27570 Germany

## Abstract

A series of multibeam bathymetry surveys revealed the emergence of a large pockmark field in the southeastern North Sea. Covering an area of around 915 km^2^, up to 1,200 pockmarks per square kilometer have been identified. The time of emergence can be confined to 3 months in autumn 2015, suggesting a very dynamic genesis. The gas source and the trigger for the simultaneous outbreak remain speculative. Subseafloor structures and high methane concentrations of up to 30 *μ*mol/l in sediment pore water samples suggest a source of shallow biogenic methane from the decomposition of postglacial deposits in a paleo river valley. Storm waves are suggested as the final trigger for the eruption of the gas. Due to the shallow water depths and energetic conditions at the presumed time of eruption, a large fraction of the released gas must have been emitted to the atmosphere. Conservative estimates amount to 5 kt of methane, equivalent to 67% of the annual release from the entire North Sea. These observations most probably describe a reoccurring phenomenon in shallow shelf seas, which may have been overlooked before because of the transient nature of shallow water bedforms and technology limitations of high resolution bathymetric mapping.

## Introduction

Pockmarks are morphological expressions of vigorous fluid escape from subaqueous sediments. Since first detections in the 1970’s and the coining of the term^1^, pockmarks have been documented in lakes^2^, estuaries^3^, on continental shelves^4^ as well as in coastal^5^ and deep sea environments^6, 7^. The bedforms are described as cone-shaped craters in the seabed^8, 9^ with diameters in the order of centimeters up to 100 s of meters and depths from a few decimeters to 10 s of meters^7, 9–11^. Their morphological expression varies from isolated elliptical features to coherent clusters or strings. The emergence of pockmarks and associated fluid seepage influences the entire local environment^12^, especially seabed flow structures^13^, morphodynamics^14^, biogeochemistry^15^ and ecology^16^. Many occurrences of pockmarks can be linked to the seepage of fluids including carbon dioxide and groundwater but the majority of reported pockmarks have been related to the expulsion of biogenic methane from the microbial degradation of organic matter^9, 17^.

In contrast to continuous diffusive seepage, the presence of pockmarks indicates a more vigorous escape of fluids from the seabed. The formation mechanism of gas induced pockmarks can be divided into three phases^8^: During the first phase, pressure is built up as gas rises from deeper reservoirs and accumulates below the seabed. When the interstitial gas pressure exceeds the load of the overlying sediment and water column, the gas erupts locally, suspending sediment into the water column and a crater remains at the eruption site. The post-eruption phase can either be a period of continuous seepage through the created vents or a dormant period until the critical pressure is exceeded again.

Identified triggers for the eruption of pockmarks include earthquakes^18, 19^, tides^20^ and storm waves^12^. In shallow water, pressure oscillations from waves of sufficient height and wave length may penetrate into the sediment to a depth equal to the wave amplitude^21^. The cyclic loading and unloading of pressure leads to regular compaction and extraction of the subseafloor inducing a pumping effect and allowing gas bubbles to rise through the sediment^8^. Finally, the release of pressure under a wave trough may lead to over-stressing and mechanical failure of the sediment matrix.

The sedimentological setting of pockmark fields is often characterized by a cohesive, clay-rich surface sediment^17^ which is also considered a good recording medium for the pockmarked morphology^10^. Pockmark dimensions scale with the characteristic grain size of the seafloor sediments^22^. Larger and deeper pockmarks with diameters in the order of 100 meters and depths in the order of 10 meters have been found in fine-grained sediment^9^. Pockmarks in coarser, e.g. sandy sediments are usually in the order of 10 m in diameter and less than 1 m in depth^9, 23^.

In the North Sea, pockmarks have previously been reported on the Dutch, the Danish, the UK and Norwegian shelf, along the Norwegian continental margin and in the Norwegian trench^9, 12, 17, 22, 24–26^. Most of these occurrences are located in deeper waters and fine grained seafloor sediments. In the German North Sea sector, no occurrences of pockmarks have been reported to date.

## The Helgoland Reef pockmark field

**Figure 1:**
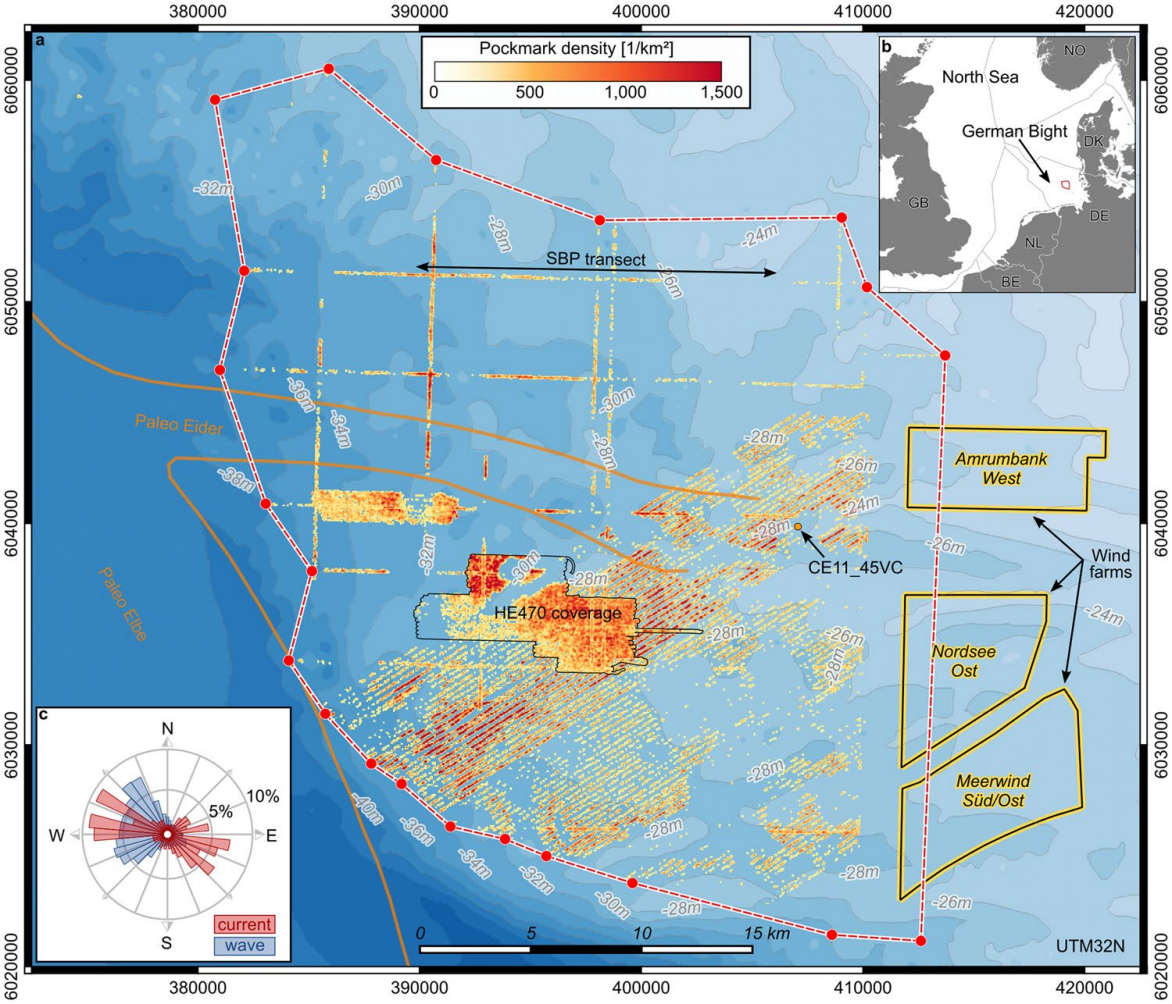
The Helgoland Reef pockmark field. (**a**) Extent of the field and pockmark density in relation to the course of the Paleo Eider and Paleo Elbe valley^29^. The location of the sub-bottom profiler (SBP) transect and location of core CE11_45VC from Fig. 4 are indicated. (**b**) Location of the Helgoland Reef pockmark field in the North Sea. (**c**) Histogram of the hydrodynamic climate at Helgoland Reef. The data were provided by the COSYNA system^34^ operated by Helmholtz-Zentrum Geesthacht Zentrum für Material- und Küstenforschung GmbH. The maps in this figure were generated using QGIS Version 2.14.11^43^. Bathymetry data was made available by the GPDN project^44^. Maritime boundaries and wind farm polygons were made available by the EMODnet Human Activities project^45^, funded by the European Commission Directorate General for Maritime Affairs and Fisheries. Wind farm data were collected by the OSPAR Commission. Maritime boundaries were provided by the European Environment Agency. Land polygons ©OpenStreetMap contributors^46^ (available under the Open Database License; see www.openstreetmap.org/copyright).

The Helgoland Reef area is located around 45 km northwest of Helgoland Island in the southeastern German Bight of the North Sea (Fig. 1a,b and Supplementary Fig. S1). Water depths range from 25 m to 40 m. The seafloor sediments consist largely of fine to medium sands with low mud content (<5%). Occasionally, the seabed exhibits mobile ripples of centimeter amplitude, indicating a morphodynamically active environment^27, 28^. Toward southwest, the area is delimited by the deeper channel of the Elbe estuary with water depths of up to 45 m and with increasing mud content. The post-glacial (10,000–8,700 y BP) confluence of the rivers Eider and Elbe was located in this region as indicated by seismo-acoustic records and drilling cores^29–31^ (Fig. 1b).

**Figure 2:**
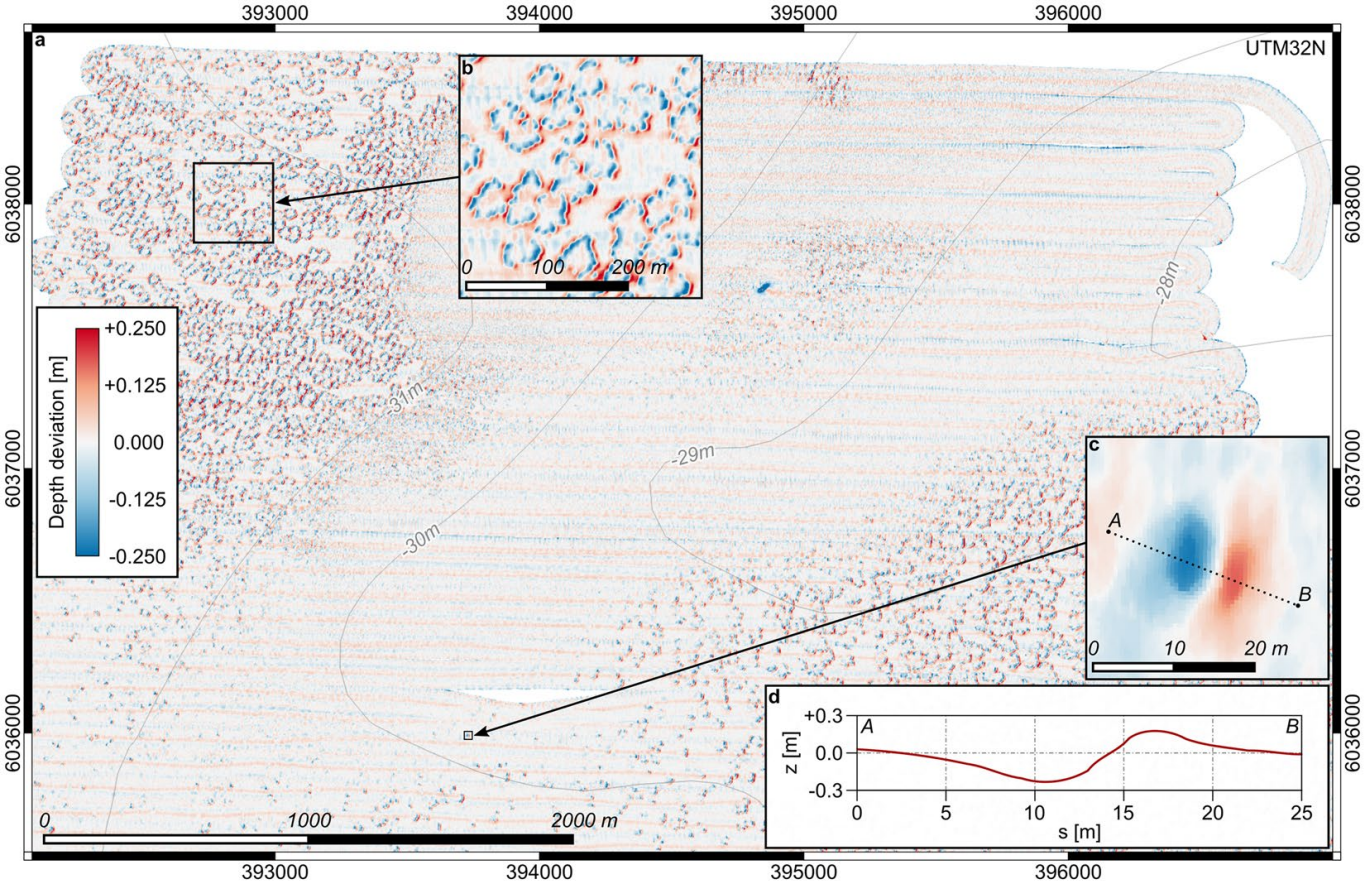
Pockmark density and morphology. (**a**) Zero-median bathymetry with full seafloor coverage from cruise HE470, August 2016. (**b**) Detail of pockmark cluster. (**c**) Detail of an individual pockmark. (**d**) Cross section along transect A–B. The maps were generated using QGIS Version 2.14.11^43^. Depth contours were made available by the GPDN project^44^.

Successive surveys with multibeam echosounder (MBES) between 2013 and 2016 revealed that the formerly flat and largely featureless sandy seafloor of the Helgoland Reef was transformed into an extensive pockmark field between July and November 2015 (see Supplementary Figs S2 and S3). When the area was surveyed after the first fall storms in November 2015, the seafloor was densely covered with elliptical depressions of around 10 m by 20 m horizontal extent and a maximum depth of around 0.2 m with respect to the surrounding bathymetry. Each depression was accompanied by a neighboring mound of similar shape and amplitude (Fig. 2c,d).

When the site was revisited in August 2016, an area of 34.25 km^2^ was surveyed with MBES with full seafloor coverage (Figs 1a and 2a). 15,506 pockmarks were detected in the digital depth models (DDMs). The depressions cover about 6% of the surveyed seafloor area. The average area of an individual pockmark depression is 140 m^2^ and the average volume is 17 m^3^. The average areal density is 453 pockmarks per square kilometer while local clusters exhibit densities of up to 1,200.

The gross volume of relocated sediment from the detected pockmark depressions amounts to around 260,000 m^3^. To delimit the overall extent of the pockmarked area, MBES surveys were continued in a wider spaced grid (Fig. 1a). The overall pockmark region covers around 915 km^2^ in water depths ranging from 25 m to 39 m. The areal density of pockmarks increases with the local water depth. Although dense accumulations are found in different absolute depths, local depressions and channels in the bathymetry exhibit denser agglomerations than mounds and ridges which are often completely free from pockmarks. Extrapolating the average pockmark density to the extended pockmark region amounts to a total of more than 410,000 pockmarks, with a gross volume of around 6,900,000 m^3^ of relocated sediment equaling about 12,000,000 t of sand (assuming a quartz sand bulk density of 2,650 kg/m^3^ and a porosity of 0.35).

## Hydro-acoustic evidence for shallow gas and active seepage

**Figure 4:**
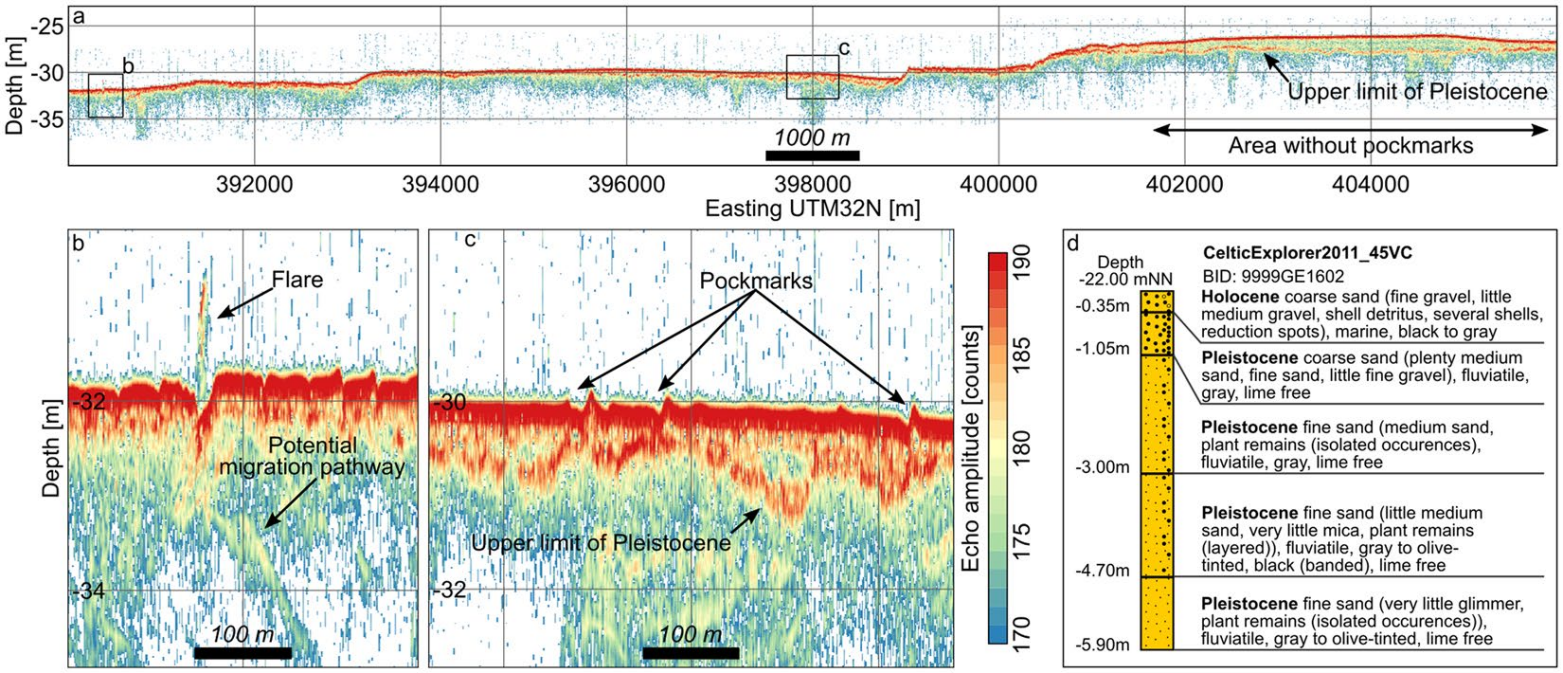
Geological setting of Helgoland Reef. (**a**) Sub-bottom profiler (SBP) transect across the pockmark field. (**b**) Detail of the methane flare. (**c**) Exemplary detail of pockmarks. (**d**) Description of core CE11_45VC^32^. See Fig. 1 for the location of the SBP transect and the core.

Shallow subseafloor records of a sub-bottom profiler (SBP) indicate potential migration pathways facilitating the ascent of the gas from shallow reservoirs (Fig. 4b). Nine months after the presumed outbreak in autumn 2015, hydro-acoustic evidence for active seepage of gas was found only for a single pockmark. A two meter high flare was identified in both frequencies (LF: 8 kHz and HF: 100 kHz) of the SBP (Fig. 4b). In long transects crossing the pockmark field, a strong reflector located a few meters below the seafloor was commonly observed in the low frequency of the SBP (Fig. 4a,c). Drilling cores suggest that it indicates the transition from the Holocene to the Pleistocene sequence^32^ (Fig. 4d). No pockmarks were found where the Holocene layer exceeded a critical thickness of around one meter (Fig. 4a).

## Pockmark morphology

The individual pockmarks consist of a well-defined crater with a neighboring mound (Fig. 2c,d). The majority of the mounds is located southeast of their associated troughs i.e., in the lee of the flood current and the dominant wave direction. A smaller part is located northwest i.e., in the lee of the ebb current (Fig. 1c). The plan of individual features is elliptical with the semi-major axis oriented northeast. Especially in regions of high areal density, the pockmarks appear in coherent ring-like structures and elongated troughs (Fig. 2b).

Backscatter intensity from MBES indicates zones of higher reflectivity inside the depressions, so called eyed pockmarks^12^. In underwater images, these zones were identified as accumulations of shell detritus. In contrast, the mounds consist of well-sorted fine sand virtually free from any shell detritus. This visual evidence supports a vigorous eruption process with subsequent settling of the suspended material as a function of grain size in the respective wave or current lee direction.

## Methane concentrations in the sediment and in the water column

Within the pockmarked area, dissolved methane concentrations of 11.0 *μ*mol/l to 30.4 *μ*mol/l were detected in the pore water and overlying bottom water samples taken from sediment cores (Supplementary Table S1). These values are around ten times higher than reference concentrations outside the pockmark field in the southeastern German Bight where values between <0.1 *μ*mol/l and 4.4 *μ*mol/l were measured.

Gas measurements in the water column were carried out using a membrane-inlet mass spectrometer (MIMS). Seawater from 25 m water depth was pumped on board and continuously analyzed with the MIMS but no significant change of carbon dioxide or methane could be associated with pockmark locations, suggesting that gas seepage had ceased at the time of the survey.

## Potential preconditions and trigger mechanisms for pockmark formation

**Figure 3:**
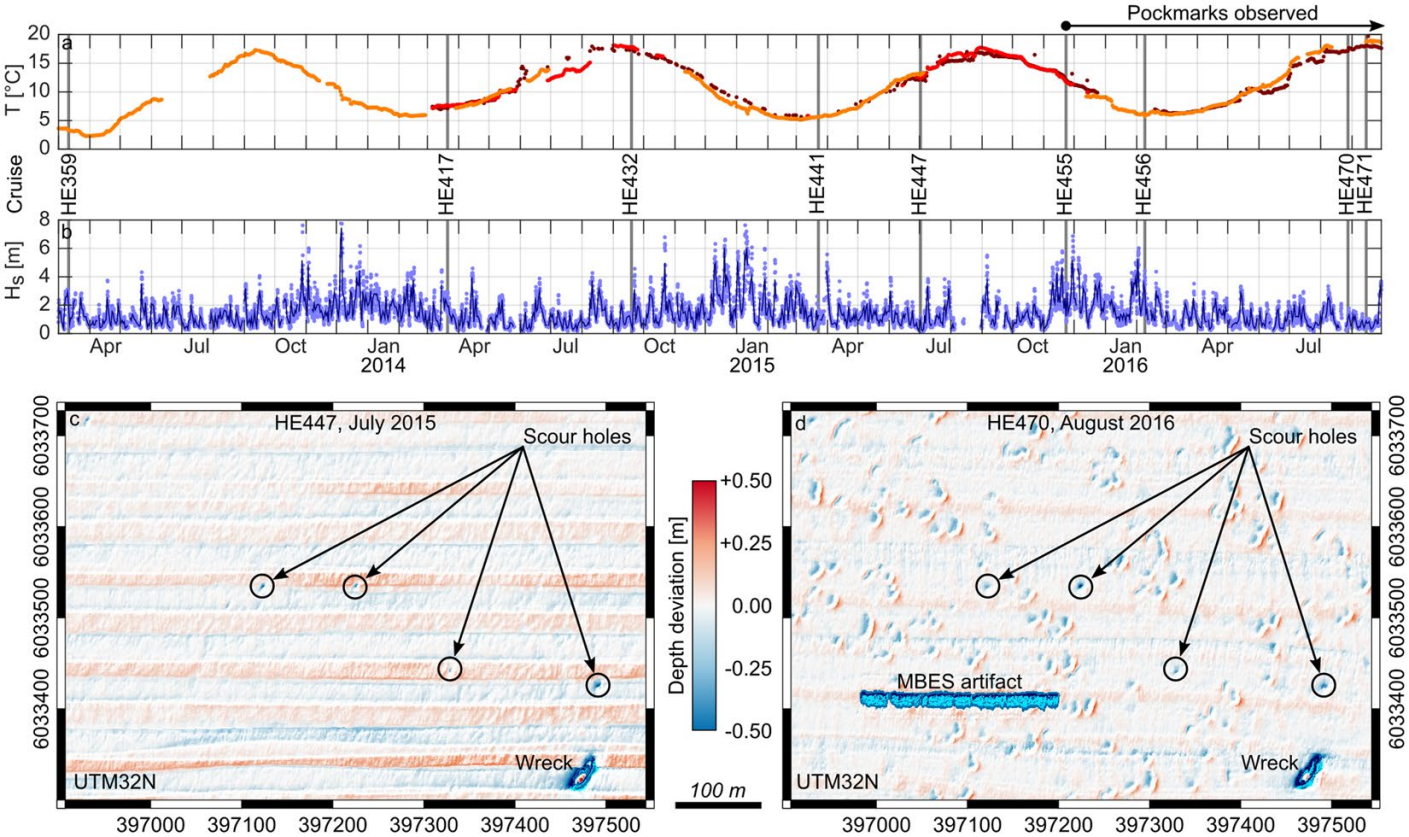
Emergence of the Helgoland Reef pockmarks. (**a**) Time series of bottom water temperature records from three stations in the German Bight. (**b**) Time series of significant wave height at Helgoland Reef from model hindcast. The data were provided by the COSYNA system^34^ operated by Helmholtz-Zentrum Geesthacht Zentrum für Material- und Küstenforschung GmbH. (**c**,**d**) Zero-median bathymetries showing the emergence of the pockmarks. For the complete record of MBES bathymetries see Supplementary Fig. S3. The maps in this figure were generated using QGIS Version 2.14.11^43^.

The most probable gas source is biogenic methane from the microbial decomposition of wetland plant remains often found in the post-glacial river confluence of ancient rivers Eider and Elbe (Fig. 4d). Comparatively high bottom water temperatures in 2014 and 2015 (Fig. 3a) may have facilitated its ascent toward a shallow depth beneath the seafloor where it remained in an unstable state until it was released by a final trigger. The region is not affected by earthquakes, but man-made tremors were generated during the pile-driving works for the construction foundations of three offshore wind farms at the eastern end of the pockmark field between 2012 and 2014 (Fig. 1a). There are no records of magnitude of the vibrations on the seafloor but the energy of the blows is considered too low and presumably dampened too fast i.e., exponentially with increasing distance from the source^33^ to cause an ascent of gas as far as 30 km away from the wind farms.

The proposed trigger for the final outbreak of the gas from the shallow subsurface is a series of storms in November 2015 (Fig. 3b). Wave model hindcasts indicate significant wave heights exceeding 7 m in the pockmarked field^34^. Typical wave peak periods measured during the winter storms in this area are in the order of 8 s to 12 s. Following linear wave theory, this results in a wave length *L* of 96 m to 177 m for an average water depth *d* of 30 m and transitional conditions (0.05 < *d*/*L* < 0.5). The effect of wave orbital motion and pressure oscillations reaches depths of 48 m to 89 m. The surface pressure oscillations and the horizontal component of the orbital velocity are reduced to around 48% by wave attenuation at a depth of 30 m. Assuming a Raleigh distribution for the wave height spectrum, the highest 1% of the waves reach 11.7 m and a maximum wave height of 14 m is possible. This allows a penetration depth of the wave-induced effective stress of up to 3.5 m for the significant and 7 m for the maximum wave^21^.

From the evidence described above, the following formation mechanism for the characteristic pockmark craters and mounds can be deduced. Triggered by a relief of pressure under a passing wave trough, the stored gas erupts and ejects sediment into the water column. The suspended material then settles in the lee of wave or current direction (whichever is dominant at that time) in a distance from the eruption point as a function of grain size. While the coarser shell debris settles back into the eruption crater, the sandy fraction is settled in a well-defined mound and the fine fraction is transported further away. An alternative mechanism for the generation of the characteristic trough-mound structures is the generation of subsidence depressions after the gas is released and a generation of the mounds as secondary sorted bedforms from the initial defects^35^. Measured near-bottom current velocities measured reach 0.3 ms^−1^ during ebb and 0.4 ms^−1^ during flood. The resulting shear stresses are capable of moving the seafloor sediment and of generating small scale bedforms with centimeter height and decimeter length scales. The dimensions and morphology of the pockmarks however are different from the typical triangular bedform cross-sections. Furthermore, a number of scour holes of around 0.5 m depth found in the area throughout all MBES surveys do not indicate any lateral mounds (Fig. 3c,d). Therefore, this formation mechanism is unlikely.

The storm events in fall 2015 that must have triggered the eruption of the pockmark field were not exceptionally extreme events. Wave heights of equal amplitude also occurred in the winters of 2013–2016 (Fig. 3b), but no pockmarks were found in the respective subsequent MBES surveys (see Supplementary Fig. S3). Although pockmarks have been observed for the first time in November 2015, it can be assumed that their emergence is a reappearing phenomenon. Following the release of the potential energy stored by the gas beneath the seafloor due to a storm event, a certain recovery period may be required to accumulate enough gas to create a new instable state. In the meantime, the shallow pockmarks as morphological symptoms of the gas release are leveled by wave and current action on the mobile sands.

As the exact date of the eruption cannot be determined, the recent morphology of the pockmarks depicts the combined effect of gas expulsion and successive scouring of the initial defects in the seafloor. While individual features can be traced throughout the calm period between February and August 2016, there is no overlap of pockmark morphologies from surveys at the beginning (HE455, Nov. 2015) and at the end (HE456, Feb. 2016) of the stormy season. The latest observed extent and the distribution of pockmarks within the field may be controlled by (a) the extent and local source strength of the presumed methane reservoir, (b) the thickness of the overlying Holocene layer and porosity and permeability of the sediment, (c) the absolute water depth as lower limit for wave impact and (d) the local variation of water depth and slope of the bathymetry providing exposure toward or shelter from wave and current action.

As this is the first description of pockmark emergence in the Helgoland Reef area, future surveys will have to shed light on the fate of the pockmarks after seepage has ceased and possible recurrence cycles of this phenomenon.

## Estimation of released gas volume

The presumed trigger mechanism suggests that the eruption of the gas occurred in a short period of time and, except for a single pockmark, ongoing continuous seepage was not observed. For a conservative estimate of the volume of the released methane, it is assumed that no cavities but only the pore space of the relocated sediment was entirely filled with gas. This may define the upper physical limit of gas stored before eruption of the pockmarks. Assuming a density of 2.067 kg/m^3^ (at 10° C and 30 m water depth) and a porosity of 0.35, the estimated gas phase removed with the relocated sediment amounts to around 5,000 t of methane. This is equivalent to 67% of the previously estimated annual methane flux from the entire North Sea (ca. 7,500 t/yr)^36^.

Methane seepages are often reported in water depth larger than 100 m and under stratified conditions^17^. Such settings extend the period of availability of the gas for methane oxidizing bacteria and archaea which delays diffusive emission to the atmosphere^37^. Due to the shallow water depth and the energetic conditions at Helgoland Reef a large amount of the methane released from the subseafloor must have been emitted to the atmosphere^38^.

An exact assessment of the marine contribution to atmospheric methane emissions is pending^36^. High methane concentrations recently observed in coastal waters^38^ may indicate an additional source of marine methane that has been neglected so far. Shallow methane reservoirs may be abundant in the post-glacial lowlands in the southern North Sea and other comparable shelf sea and coastal environments with organic-rich deposits worldwide^23, 39, 40^. However, the abrupt and simultaneous emergence of more than 300,000 pockmarks in less than five months has not been reported so far. A possible explanation is that pockmarks, like other bedforms, are transient features especially in shallow and morphodynamically active shelf areas. The short appearance of pockmarks may not match the frequency of bathymetric surveys. In addition, the detection of the 0.2 m shallow pockmarks at the Helgoland Reef was only possible due to recent advances in mapping technology^41, 42^. Similar pockmark fields in coastal areas and on continental shelves may have been overlooked to date.

## Methods

### Bathymetry mapping and pockmark detection

The bathymetric surveys were conducted with a Kongsberg EM 710 multibeam echo sounder coupled with differential GPS positioning onboard R/V Heincke. Raw data were processed and gridded using the multibeam processing suite MB-System. The resulting digital depth models (DDMs) were further processed using the Generic Mapping Tools (GMT) Version 4. To identify the shallow pockmark features against the larger variability in the bathymetry, a moving median filter with 50 m diameter was applied using the *grdfilter* function. The resulting background bathymetry was subtracted from the original DDMs producing zero-median DDMs. A depth contour of −0.05 m was selected to detect pockmarks in the zero-median DDMs. The resulting polygons were filtered in QGIS Version 2.14.11^43^ to remove artifacts caused by ship motion that remain after motion compensation. Features with a minimum depth of less than 0.15 m, a median depth of less than 0.075 m and an area of less than 10 m^2^ or greater than 500 m^2^ were removed. Finally, obvious remaining artifacts were manually removed after visual inspection. The centroids of the remaining polygons were used to generate heat maps of pockmark density using the *gdal*_*grid* algorithm. Points were counted within a radius of 56.4 m, equaling an area of 0.01 km^2^. The volume of relocated sediment was calculated from the zero-median DDMs using the *zonal statistics* function in QGIS with the −0.05 m contour lines as mask layer.

### MBES data quality and scour holes

Prior to the first detection of the pockmarks during cruise HE455, an area of 5.5 km^2^ had been surveyed with MBES repeatedly during cruises HE417, HE432, HE441, HE447 (Fig. 3 and Supplementary Fig. S2). The only notable morphological features, apart from a shipwreck at the bottom of a scour, were a number of circular depressions of around 0.5 m depth (Fig. 3c,d). On a recent high-resolution survey we found what we believe to be rounded boulders in their deepest points. The ability to resolve these features can be taken as proof of quality for the multibeam data. Although some of the surveys (especially HE455 and HE 456) were carried out in heavy sea state and exhibit artifacts from badly compensated ship motion, the ability to resolve the scour holes proves the reliability of the system. The holes can be found throughout all MBES surveys. When compared to the pockmarks, they are easily distinguishable as they are more circular in shape and lack the lateral mound.

### Sub-bottom profiling

Sub-bottom profiles were recorded with a parametric echosounder (Innomar SES-2000 medium) with acoustic frequencies of 8 kHz and 100 kHz. The penetration of the low-frequency signal into the sandy seafloor was around 5 m below the seabed.

### Methane detection

The sediment cores were taken with a multicorer (MUC) from the positions MUC1 to MUC6 inside the pockmark field and from several additional stations in the German Bight (Supplementary Table S1, Fig. S1). An acoustic ultra-short baseline transponder (iXblue GAPS Carbon V.1) was used to record the position of the MUC on the seafloor. Pore water samples from MUC cores were taken at a depth of 0.05 m, transferred bubble-free and without a headspace into Exetainer (5.9 ml), fixated with 100 *μ*l ZnCl (1 M) and stored cool and dark. In order to avoid methane loss or contamination, the samples were neither filtered nor was a vacuum applied during pore water sampling. Bottom water samples were taken from the overlying water of the MUC cores and treated in the same way as the pore water samples. The samples were analyzed in a membrane inlet mass-spectrometer (MIMS) under fully controlled temperature conditions. Calibration for methane concentration was carried out using standard methane mixtures (1.725 ppm (0.07 *μ*mol/l); 209.7 ppm (8.8 *μ*mol/l) and 1004 ppm (42.02 *μ*mol/l)).

During cruise HE470, MIMS measurements were performed on board measuring carbon dioxide and methane concentrations of bottom water that was pumped on deck. Due to lack of correct standard gas mixtures, only the potential changes in concentrations were studied.

### Data avaiability

Relevant data will be made available on PANGAEA^®^.

## Electronic supplementary material

Supplementary Information
