# Supplementary material for: Abrupt emergence of a large pockmark field in the German Bight, southeastern North Sea
Source: Sci Rep. 2017 Jul 11;7:5150. doi: 10.1038/s41598-017-05536-1 (PMC5505999; doi:10.1038/s41598-017-05536-1)
Supplement: Supplementary file 1 — Supplementary Information [file 41598_2017_5536_MOESM1_ESM.pdf]

Abrupt emergence of a large pockmark field in the German Bight, southeastern North Sea

Knut Krämer, Peter Holler, Gabriel Herbst, Alexander Bratek, Soeren Ahmerkamp, Andreas Neumann, Alexander Bartholomä, Justus E.E. van Beusekom, Moritz Holtappels, Christian Winter

Supplementary Table S1. Methane concentrations in pore and bottom water samples from the Helgoland Reef are and the wider German Bight during cruise HE471, September 2016

Sample	Method	C [µmol l^{-1}]
	Helgoland Reef	
Core 1	incubation	18.7
Core 2	incubation	36.5
MUC2	bottom water	30.4
MUC3	pore water	26.3
MUC4	bottom water	28.1
MUC4	pore water	11.0
MUC5	bottom water	28.8
MUC5	pore water	27.9
MUC6	bottom water	26.2
MUC6	pore water	16.8
	German Bight	
CCP-G	bottom water	4.4
CCP-G	pore water	3.0
NOAH-A	bottom water/incubation	1.0
NOAH-C	bottom water/incubation	3.2
NOAH-D	bottom water/incubation	<0.1
Tonne E3	bottom water/incubation	<0.1

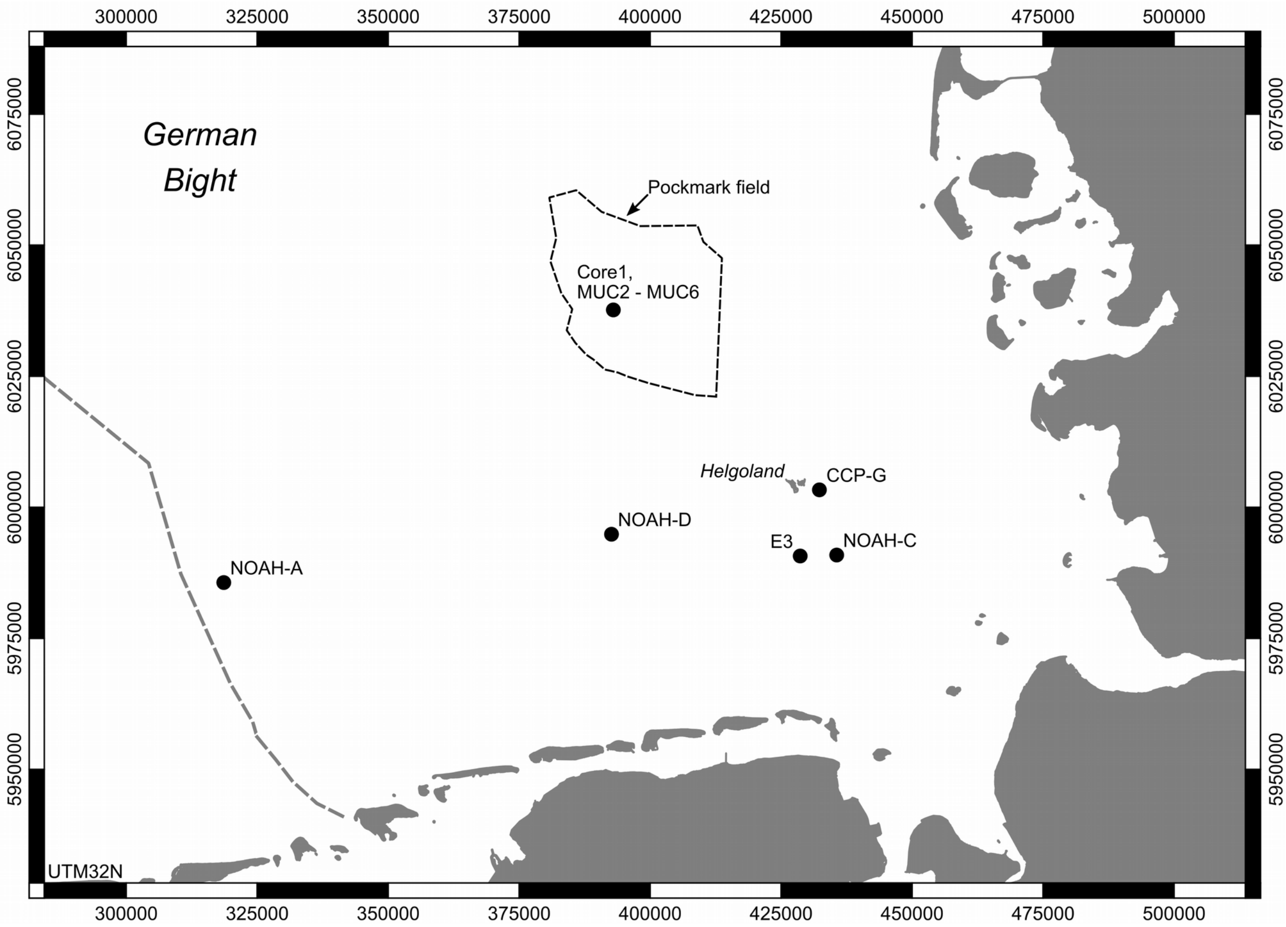

Supplementary Figure S1. Locations of sediment samples from Tab. S1. The map was generated using QGIS Version 2.14.11[1]. Maritime boundaries were made available by the EMODnet Human Activities project[2], funded by the European Commission Directorate General for Maritime Affairs and Fisheries. Data were provided by the European Environment Agency. Land polygons ©OpenStreetMap contributors[3] (available under the Open Database License; see www.openstreetmap.org/copyright).

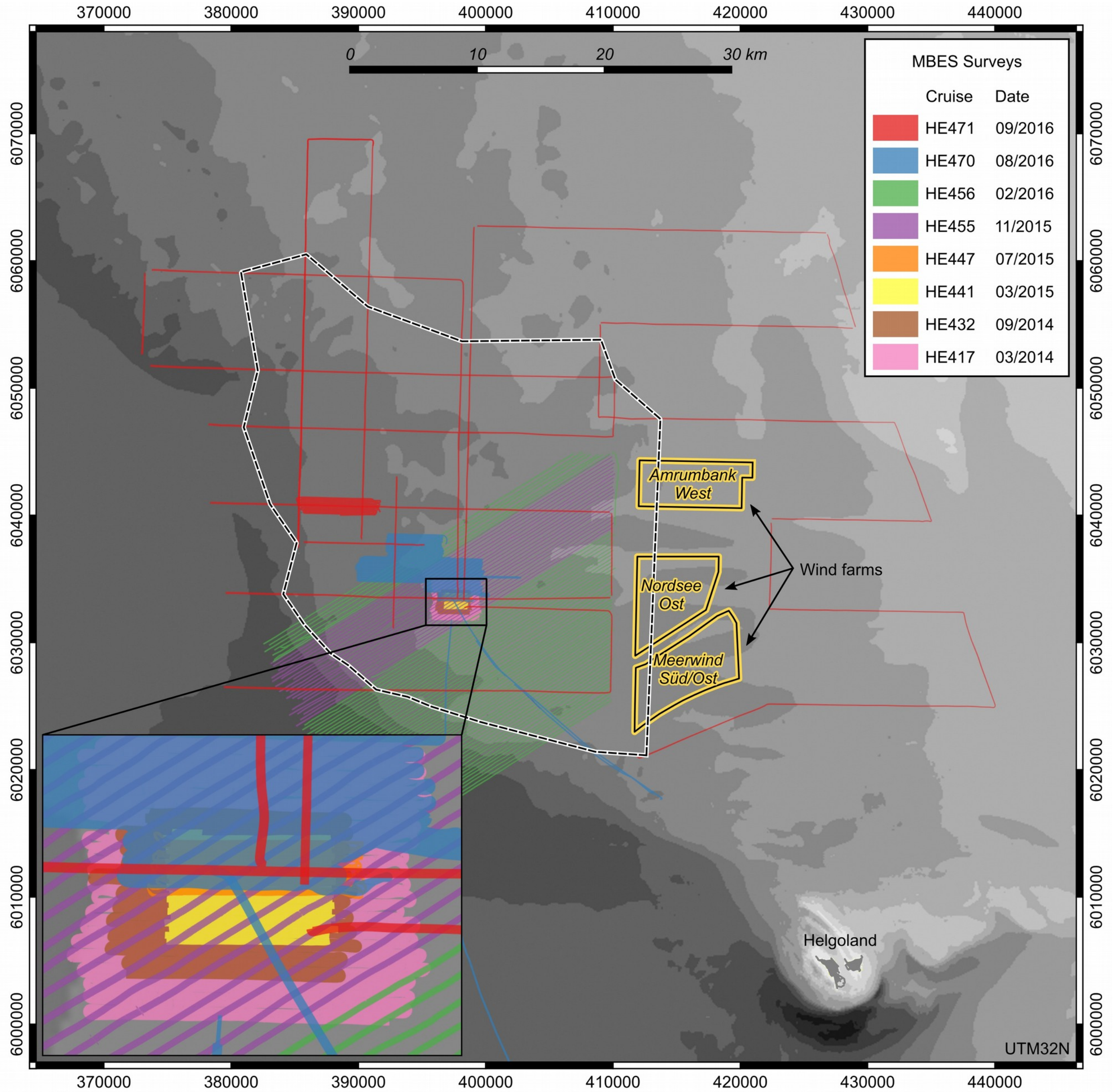

Supplementary Figure S2. Overview of MBES surveys. The seafloor coverage of MBES swaths is given in different color coding for the respective cruises. The map was generated using QGIS Version 2.14.11[1]. Wind farm polygons were made available by the EMODnet Human Activities project[2], funded by the European Commission Directorate General for Maritime Affairs and Fisheries. Wind farm data were collected by the OSPAR Commission. Bathymetry data were made available by the GPDN project[4]. Land polygons ©OpenStreetMap contributors[3] (available under the Open Database License; see www.openstreetmap.org/copyright).

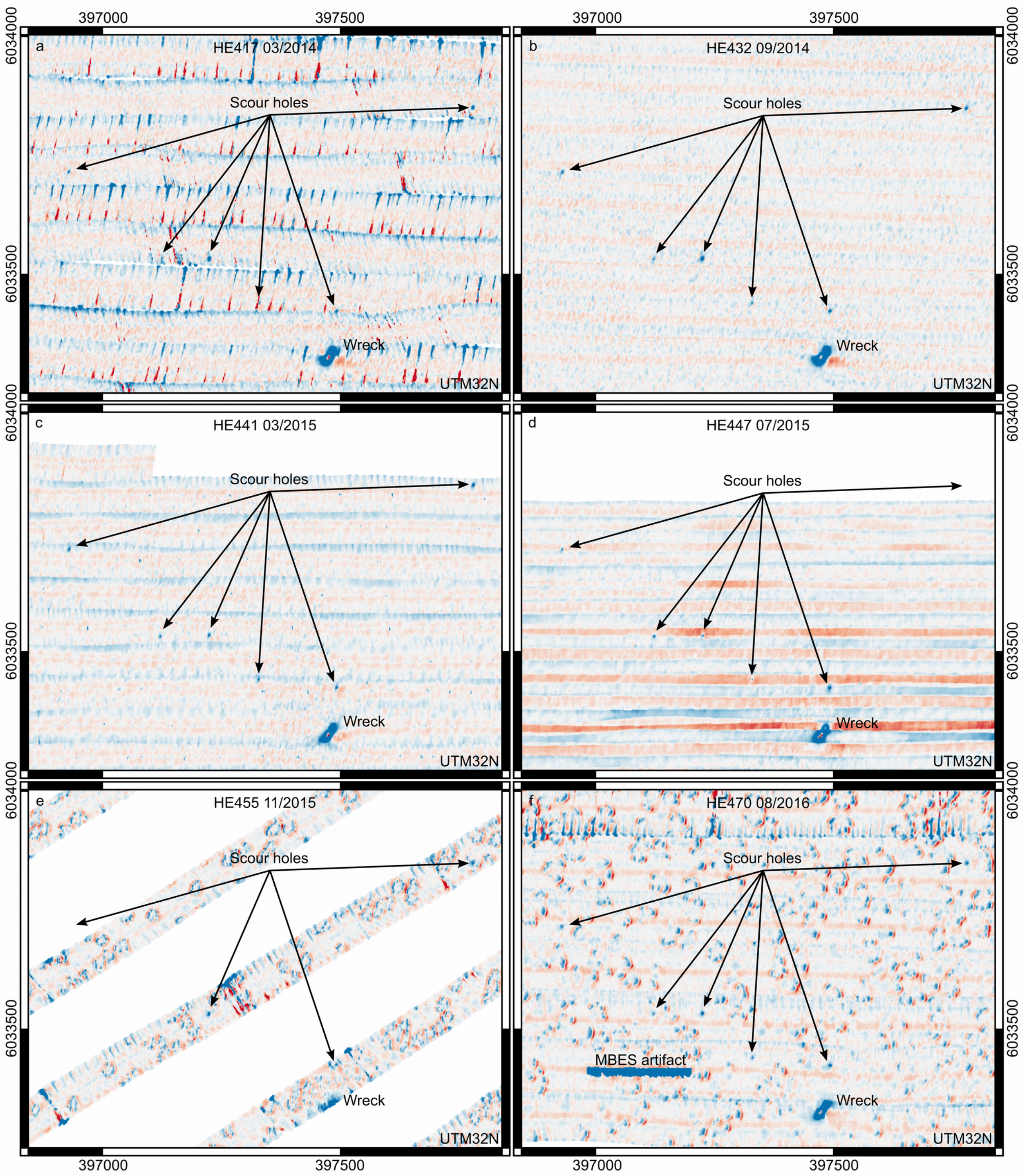

Supplementary Figure S3. Seafloor evolution and appearance of pockmarks. Pockmarks were first observed during cruise HE455 in November 2015 (e). In this area, they have changed in location and morphology when last observed during cruise HE470 in August 2016 (f). Some features are persistent: In the lower part of the figure the scour around a small ship wreck can be located as well as a few other small scour features possibly connected to boulders on the seafloor. The maps in this figure were generated using QGIS Version 2.14.11[1].

References

1. QGIS Development Team. QGIS Geographic Information System (2016). URL http://qgis.osgeo.org.

2. European Marine Observation and Data Network (EMODnet). EMODnet Human Activities (2017). URL http://www.emodnet-humanactivities.eu.

3. OpenStreetMap contributors. Planet dump retrieved from https://planet.osm.org (2016). URL https://www.openstreetmap.org.

4. Geopotential Deutsche Nordsee (GPDN). Bathymetrie, Intervalle mit 1 m und 5 m Abstand (2013). URL http://www.gpdn.de/
